# Mesenchymal stem cell-derived extracellular vesicles for human diseases

**DOI:** 10.20517/evcna.2023.47

**Published:** 2024-02-06

**Authors:** Xiaofang Zhang, Xiaofang Che, Sibo Zhang, Runze Wang, Mo Li, Yi Jin, Tianlu Wang, Yingqiu Song

**Affiliations:** ^1^Cancer Hospital of China Medical University, Liaoning Cancer Hospital and Institute, Cancer Hospital of Dalian University of Technology, Faculty of Medicine, Dalian University of Technology, Shenyang 110042, Liaoning, China.; ^2^Department of Medical Oncology, The First Hospital of China Medical University, Shenyang 110001, Liaoning, China.; ^3^The Fourth Hospital of China Medical University, Shenyang 110032, Liaoning, China.; ^#^Authors contributed equally.

**Keywords:** Mesenchymal stem cell, extracellular vesicles, inflammation, tumor, immunity, tissue repair

## Abstract

Stem cell therapy is a novel approach for treating various severe and intractable diseases, including autoimmune disorders, organ transplants, tumors, and neurodegenerative diseases. Nevertheless, the extensive utilization of stem cells is constrained by potential tumorigenicity, challenges in precise differentiation, rejection concerns, and ethical considerations. Extracellular vesicles possess the ability to carry diverse bioactive factors from stem cells and deliver them to specific target cells or tissues. Moreover, they offer the advantage of low immunogenicity. Consequently, they have the potential to facilitate the therapeutic potential of stem cells, mitigating the risks associated with direct stem cell application. Therefore, the use of stem cell extracellular vesicles in clinical diseases has received increasing attention. This review summarizes advances in the use of extracellular vesicles from mesenchymal stem cells (MSC). MSC extracellular vesicles are used in the treatment of inflammatory diseases such as rheumatoid arthritis, liver injury, COVID-19, and allergies; in the repair of tissue damage in heart disease, kidney injury, and osteoarthritic diseases; as a carrier in the treatment of tumors; and as a regenerative agent in neurodegenerative disorders such as Alzheimer's and Parkinson's.

## INTRODUCTION

Mesenchymal stem cells (MSCs) are multipotent non-hematopoietic adult stem cells derived from various tissues such as bone and adipose, commonly referred to as pluripotent stromal cells or mesenchymal stromal cells^[1]^. MSCs possess numerous advantageous properties, including low immunogenicity, chemotaxis, and the ability to target tumor sites with precision^[2]^. They communicate with target cells, thereby influencing the activity and function of these cells, playing pivotal roles in tissue repair and disease treatment^[3]^. However, the widespread application of stem cell transplantation has been limited due to safety and ethical concerns. Consequently, an increasing number of studies are being conducted on extracellular vesicles (EVs)^[4]^. EVs exhibit characteristics and biological activities similar to MSCs but also possess advantages such as targeted delivery, low immunogenicity, and high repairability. Furthermore, EV-based treatments follow a safe, cell-free therapy strategy^[5,6]^. MSC-EVs serve as messengers between cells and can regulate various physiological processes. They influence disease progression by carrying bioactive factors (including proteins, lipids, mRNA, and miRNA)^[7-9]^. Therefore, MSC-EVs can be employed as natural drug delivery vehicles with therapeutic effects for a wide range of diseases^[10]^. Additionally, MSC-EVs exhibit anti-inflammatory, immunomodulatory, and regenerative repair activities^[11]^. This article provides an overview of the utilization of MSC-EVs in numerous diseases, including rheumatoid arthritis, liver injury, COVID-19, hypersensitivity reactions, heart disease, kidney injury, osteoarticular disease, cancer, Alzheimer's disease, and Parkinson's disease [Table 1].

**Table 1:** MSC-Exo treats multiple diseases

**Function**	**Disease**	**Exo**	**Effect**	**Reference**
Anti-inflammatory	RA	miR-135b	Promote chondrocyte proliferation	[18]
		miR-150-5p	Alleviate abnormal vascular proliferation	[18]
	Liver injury	Hepatocyte growth factor	Promote hepatocyte growth	[20]
	COVID-19	Exo- Liposome	Inhibit viral intracellular pathways and suppress inflammatory cytokine storm	[32,33]
	Hypersensitivity	ovalbumin	Modulate the immune response	[36]
		miR-146a-5p	Alleviate airway hyperresponsiveness	[38]
Tissue repair	Heart disease	miR-24-3p	Reduce myocardial infarction	[54]
		miR-183-5p	Inhibit cardiomyocyte aging	[56]
		miR-146a-5p	Attenuate drug-induced oxidative stress in cardiomyocytes	[59]
		IONP-Exo	Reduce cardiomyocyte apoptosis and fibrosis	[64]
	Kidney damage	miR-186-5p	Inhibit ECM protein accumulation and epithelial-mesenchymal transition and attenuates renal fibrosis	[70]
		miR-146b	Reduce IL-1 receptor-related kinase expression and inhibit NF-κB activity	[73]
	Osteoarthritis	VEGF	Enhance angiogenesis	[78]
		CLEC11A	Enhance bone formation and reduce bone resorption	[79]
		CD73	mediate cartilage repair and regeneration	[80]
		miRNA-128-3p	Target inhibition of Smad5 attenuates cellular osteogenic differentiation and fracture healing *in vivo*	[83]
Anti-tumor factor vector	CRC	miR-16-5p	Down-regulate integrin α2 and inhibit cancer cell progression	[84]
		miR-326	Inhibit NF-κB and thus reduce immune factors	[86]
		miR-1246	Restore Th17/Treg balance and reduce IBD	[88]
	HNSCC	miR-101-3p	Inhibit cancer cell proliferation and tumor growth	[93]
		siRNA	Knockdown of Survivin gene to inhibit tumor growth	[95]
Regeneration	AD	GDF-15	Mitigate Aβ42-induced apoptosis and inflammation	[111]
	PD	peroxisome mRNA	Reduce neurotoxicity and neuroinflammation	[118]

RA: rheumatoid arthritis; COVID-19: Coronavirus disease 2019; IONP: iron oxide nanoparticles; ECM: extracellular matrix; IL: interleukin; CRC: colorectal cancer; IBD: inflammatory bowel disease; HNSCC: head and neck squamous cell carcinoma; AD: Alzheimer's disease; GDF: growth differentiation factor 15; PD: Parkinson's disease.

## MSC-EVS FOR INFLAMMATION-RELATED DISEASES

MSCs secrete EVs that possess therapeutic effects in inflammation-related diseases^[12]^. When EVs are isolated from MSC, modified by specific miRNA therapeutic agents, and then injected into the body via intravenous injection, the enclosed miRNAs can then be transferred from the EVs to specific recipient cells. For example, in diabetic mouse models, EV-miRNAs complexes interact with the Toll-like receptor (TLR) signaling pathway to regulate the activation of NF-κB, which in turn produces more immune factors, such as interferon-alpha (IFN-α), tumor necrosis factor-alpha (TNF-α), interleukin-1beta (IL-1β), and interleukin-6 (IL-6), and thus affects the disease status. Moreover, MSC-EVs significantly inhibit pro-inflammatory cytokines and contain multiple miRNAs targeting the Toll-like receptor 4/NF-κB signaling pathway^[13]^. This reduction in inflammatory cytokines production is achieved by inhibiting tumor necrosis factor receptor-associated factor 6 and IL-1 receptor-associated kinase 1 expression, particularly following macrophage polarization induction towards the M2 phenotype via the administration of EVs containing miR-146a. These findings corroborate the therapeutic efficacy of the EV-miRNA complex in the context of inflammatory diseases^[14]^. Consequently, MSC-EVs are capable of exerting anti-inflammatory effects during disease progression.

### Rheumatoid arthritis

Rheumatoid arthritis (RA) is a chronic systemic disease involving autoimmunity, characterized by inflammatory changes in synovial and joint structures, extensive fibrinoid degeneration of collagen fibers in the mesenchymal tissue, and atrophy and thinning of bone structures. Currently, more than 30% of patients with RA exhibit an inadequate response to first-line treatment, with joint replacement being the only available treatment option for those with advanced RA^[15]^. Therefore, there is an imperative need for novel therapeutic strategies to enhance the efficacy of RA treatment.

In animal experiments, canine MSC-derived EVs exhibit anti-inflammatory and immunomodulatory effects *in vitro* under serum-free medium conditions^[16]^. Feline adipose tissue-derived MSC-EVs demonstrate an increased expression of IL-10 (an anti-inflammatory factor) and a significant decrease in pro-inflammatory factor expression^[17]^. These findings underscore the potential of MSC-EVs in the treatment of inflammatory diseases.

In the following sections, we will discuss the role of microRNAs carried by MSC-EVs in RA. Enhanced expression of MSC-EV-derived miR-135b, stimulated by transforming growth factor-β1, inhibits the expression of specific protein 1 in chondrocytes, thereby promoting chondrocyte proliferation and facilitating the repair of damaged cartilage tissue. Additionally, MSC-EV-derived miR-150-5p reduces joint destruction by targeting matrix metalloproteinase 14 and vascular endothelial growth factor, thus ameliorating abnormal vascular proliferation and inhibiting synovial cell proliferation in RA^[18]^. However, the utility of EVs is constrained by their short half-lives. A study has demonstrated that hydrogels can seamlessly bond with natural cartilage. The hydrogel delivery of cartilage-bound EVs enhances the stability of proteins and miRNAs within MSC-EVs, prolonging their *in vivo* retention and controlled drug release. Consequently, hydrogels provide an effective scaffold for the transport of EVs to facilitate the repair and regeneration of articular cartilage defects^[19]^.

### Liver injury

EVs present a promising cell-free strategy for the treatment of liver injuries^[20]^. For instance, rat bone marrow MSC (BMSC)-EVs can expedite hepatocyte proliferation and alleviate hepatic fibrosis by inhibiting hepatocyte scorch death^[21]^. Furthermore, *in vivo* human umbilical cord-derived MSC (hUC-MSC)-EVs contribute to the repair of damaged liver tissues by reducing Nod-like receptor protein 3 inflammasome vesicle expression in macrophages within a mouse model of acute liver failure^[22]^. EVsomes play a pivotal role in combating hepatic diseases by transporting a diverse array of cargo compounds to target cells. Specifically, MSC-EVs have been employed as delivery vehicles for dexamethasone in the treatment of autoimmune hepatitis^[23]^. BMSC-EVs are a promising nanocarrier with the potential to substantially enhance the anti-fibrotic effects of lignocaine^[24]^.

### COVID-19

Coronavirus disease (COVID-19) is a global pandemic caused by severe acute respiratory syndrome coronavirus type 2 (SARS-CoV-2). SARS-CoV-2 targets mucosal cells of the respiratory system, in turn infecting other cell types. This may induce a systemic inflammatory storm that triggers an acute respiratory distress syndrome, often eventually leading to multi-organ damage^[25]^. Clinical studies have shown that MSC-EVs can significantly reduce various types of lung inflammation. MSC-EVs may contribute to the modulation of immune responses, promote pathogen clearance, and reduce the severity of organ damage. The immunomodulatory capabilities of MSCs include the inhibition of T and B cell proliferation and cytokine production, reduction of NK cell function, and maturation of dendritic cells. MSCs and their derivative EVs are potential therapeutic elements for the treatment of COVID-19^[26-28]^.

COVID-19 immunization is divided into two main phases. The first phase is the elimination of the virus and suppression of disease progression to a critical stage, and the second phase is an inflammatory state that can lead to a cytokine storm. SARS-CoV-2 enters host cells through endocytosis mediated by the angiotensin-converting enzyme 2 (ACE2) receptor. In addition to immune system dysregulation, dysfunction of the renin-angiotensin system caused by ACE2 downregulation is also related to COVID-19^[29]^. P9R is a defensin-like peptide with antiviral activity^[30]^. The positive charge of P9R effectively inhibits cytoplasmic matrix protons entering the endosome, thereby preventing endosomal acidification and impacting the life cycle of SARS-CoV-2. 8P9R is a branching form of P9R that forms cross-links with viruses to enhance antiviral activity. Chimeric 8P9R-antifungal peptide binds to SARS-CoV-2 with higher specificity and can effectively inhibit the entry of SARS-CoV-2 into target cells for replication^[31]^.

**Figure 1:**
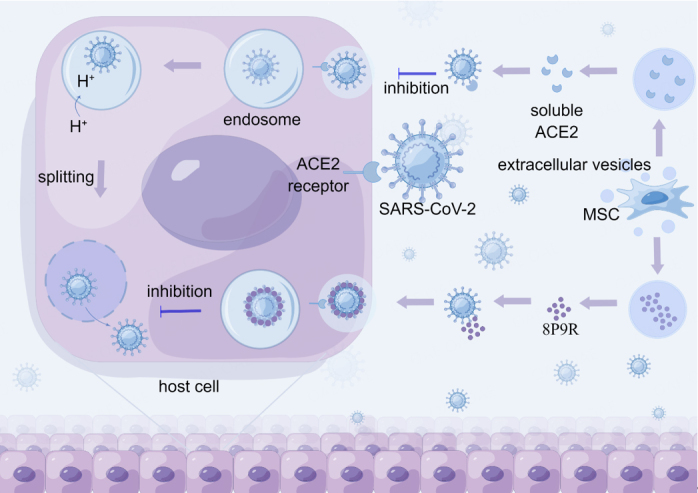
MSC-EV-hybrid liposomes inhibit the SARS-CoV-2 intracellular pathway. MSC-EVs were hybridized with synthetic liposomes to create vectors expressing chimeric 8P9R-anti-peak peptides and soluble ACE2, and administered via inhalation. The 8P9R chimeric peptide specifically enters virus-infected cells by binding the virus via ACE2-mediated endocytosis, subsequently blocking H^+^ entry into the endosome, inhibiting acidification of the endosomal environment, and preventing viral escape into the cytoplasmic matrix for replication. Soluble ACE2 acts as a decoy receptor to capture extracellular viruses and reduce the viral load, thereby inhibiting the viral intracellular pathway.

MSC-EVs were hybridized with synthetic liposomes of an appropriate size to generate a vector that encompassed the chimeric 8P9R-anti-peak peptides and soluble ACE2. The resulting therapeutic agent was administered via inhalation^[32]^. These EV-liposome hybrids can inhibit the SARS-CoV-2 intracellular pathway and suppress cytokine storms, reducing mortality in patients with COVID-19^[33]^. The process is divided into two parts: in the first phase, intracellular pathways that are critical to viral replication are inhibited by the chimeric 8P9R peptides, which enter virus-infected cells by binding specifically with the virus. Subsequently, they inhibit the acidification of the endosomal environment, preventing viral escape into the cytoplasmic matrix and inhibiting viral replication. In the second phase, the NF-κB-sensitive promoter regulates the level of soluble ACE2 and forestalls the excessive inflammatory state triggered by ACE2 downregulation in patients. Besides that, soluble ACE2 can act as a decoy receptor to competitively bind viruses and reduce viral loads^[34]^ [Figure 1]. Owing to the low immunogenicity of EVs, they can be transferred without triggering an immune response. Therefore, the preparation of EV-liposome hybrids may be a suitable therapeutic option.

### Hypersensitivity

The immunomodulatory properties and anti-inflammatory effects of MSCs are mediated through intercellular contacts and soluble factors^[35]^. In mice, for example, prophylactic use of ovalbumin-enriched MSC-derived EVs modulated immune responses, significantly decreased IgE levels and IL-4 production, increased TGF-β levels, and suppressed allergic asthma induced by ovalbumin sensitization^[36]^. Song *et al.* pretreated human umbilical cord-derived MSCs (hUC-MSCs) with IL-1β, which upregulated the expression of anti-inflammatory miR-146a, thereby effectively enhancing their immunomodulatory properties^[37]^. HMSC-EVs inhibit the levels of group 2 innate lymphocytes by delivering miR-146a-5p, thereby reducing inflammatory cell infiltration, lung mucus production, and airway hyperresponsiveness. This alleviates group 2 innate lymphocyte-dominant allergic airway inflammation, suggesting that MSC-EVs represent a novel cell-free strategy for treating inflammatory diseases^[38]^. Administration of MSC-EVs can prevent adverse complications associated with cell transplantation, such as immune rejection^[39]^. For instance, hUC-MSC-EVs can prevent life-threatening acute graft-versus-host disease (GVHD) in a mouse model of allogeneic hematopoietic stem cell transplantation^[40]^. MSC-EVs also improve survival in mouse models of chronic GVHD by inhibiting Th17 cells and inducing regulatory T cells (Treg)^[41]^, representing a novel and alternative approach for treating GVHD.

## MSC-EVS' REPAIR CAPABILITY TO TREAT TISSUE INJURIES

Stem cell-derived EVs can improve inflammation, prevent tissue damage, and promote healing, primarily through the action of RNA/miRNA^[42]^. For instance, miRNA within UC-MSC-EVs inhibits myofibroblast differentiation, ameliorates scarring resulting from myofibroblast aggregation, and promotes skin repair by inhibiting the transforming growth factor-β/SMAD2 pathway during wound healing^[43]^. UC-MSC-EVs, containing numerous growth factors such as CD63 and HSP70, when injected into the cochlea of mice with ototoxicity-induced hearing damage, lead to altered gene expression in the hair cells, increased remodeling of damaged neural tissues, decreased apoptosis, and neural damage repair^[44,45]^.

### Heart disease

**Figure 2:**
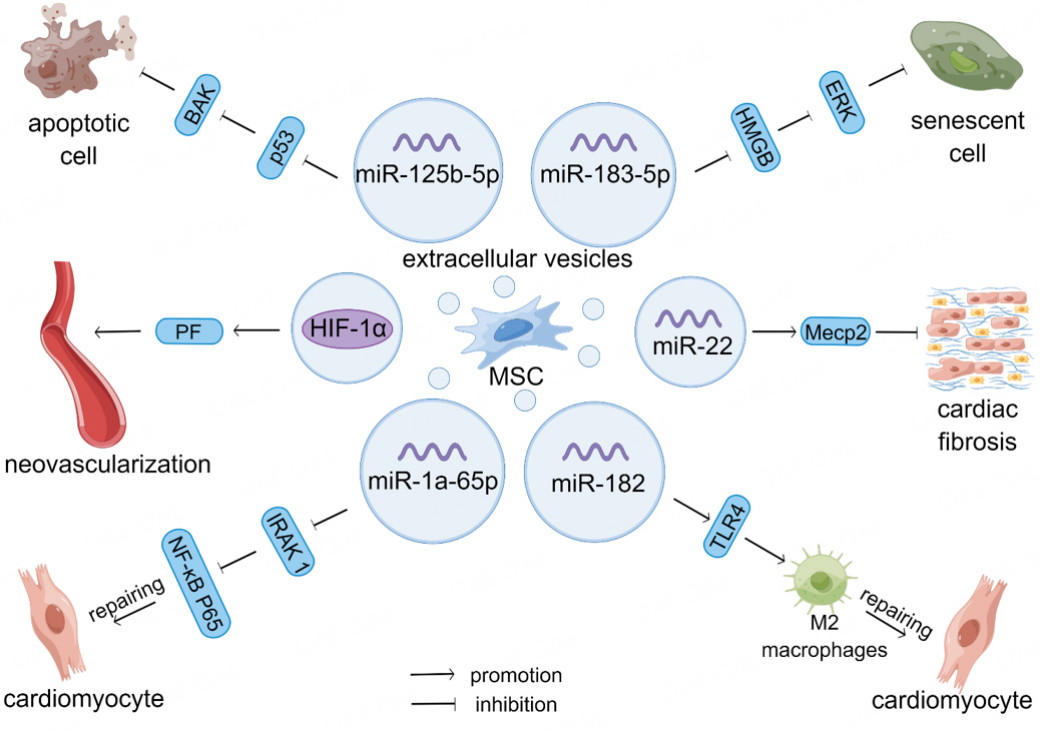
MSC-Exos carry bioactive factors involved in the repair of myocardial injury. MSC-Exos contain bioactive miRNAs that mitigate cardiomyocyte injury by participating in anti-inflammatory, anti-apoptotic, anti-aging, and promoting M2-type macrophage differentiation. MSC-Exos carry HIF-1α to inhibit cardiomyocyte fibrosis and promote neovascularization, thereby repairing myocardial injury. p53: recombinant tumor protein p53; BAK1: Bcl2 Antagonist/Killer 1; miR: microRNA; PF: platelet factor; HIF-1α: hypoxia-inducible Factor-1α; IRAK 1: interleukin-1 receptor-associated kinase 1; NF-κB: nuclear factor kappa-B; ERK: extracellular regulated protein kinases; HMGB: high-mobility group box; Mecp2: methyl CpG binding protein 2; TLR4: Toll-like receptor 4.

Heart disease has long been a category of diseases that are highly associated with death worldwide^[46]^. In the early stages of acute myocardial infarction (MI), a large number of cardiomyocytes die suddenly, accompanied by a robust inflammatory response. This results in serious clinical outcomes such as decreased cardiac function, ventricular remodeling, and heart failure^[47]^. Due to the multidirectional differentiation potential and anti-inflammatory effects of MSCs, it holds promise as a treatment for patients with acute MI^[48]^. The cardioprotective effects of MSCs are mainly mediated through their paracrine effects and the transfer of EVs rich in bioactive proteins, RNA, and lipids^[49]^. MiRNA-carrying EVs involved in the regulation of the inflammatory response and in the repair of myocardial fibrosis play important roles in myocardial infarction and injury^[50]^ [Figure 2]. EV-lncRNAs derived from MSCs prevent cardiomyocyte apoptosis *in vivo* and play a key role in the development of heart disease as novel regulators of cardiovascular disease^[51]^.

MIs are usually associated with angiogenic dysfunction. In this section, we will discuss the mechanisms by which MSC-EVs contribute to the recovery from myocardial ischemic injury. EVs from human-induced pluripotent stem cell-derived MSCs ameliorate MI injury by activating the Akt/Nrf2/HO-1 signaling pathway and increasing the expression of von Willebrand factor and vascular endothelial growth factor^[52]^. Hypoxia-inducible factor-1α overexpression by MSC-EVs increases the expression of pro-angiogenic factors in rats, reduces human umbilical vein endothelial cell (HUVEC) injury, promotes neointima formation, and inhibits fibrosis to maintain cardiac function in a rat MI model^[53]^. hUC-MSC-EVs increase miR-24-3p expression in macrophages, and miR-24-3p inhibits the activation of the NF-κB signaling pathway in inflammatory environments, thereby promoting M2 macrophage polarization and reducing MI injury^[54]^. MSC-EVs also regulate macrophage polarization to the M2 phenotype via miR-182 and attenuate myocardial ischemia-reperfusion injury^[55]^. In addition, heme-pretreated MSC-EVs (enriched with miR-183-5p) inhibited cardiomyocyte senescence by modulating the high mobility group box chromosomal protein 1/extracellular signal-regulated kinase pathway, and were superior to untreated MSC-EVs for the treatment of MI^[56]^.

The most immediate physiologic change resulting from myocardial ischemia is hypoxia. Under hypoxic conditions, BMSC-EVs carrying miR-125b-5p may downregulate the expression of p53 and BAK1 in cardiomyocytes, ameliorate cardiomyocyte apoptosis, and promote ischemic heart repair. This ability of BMSC-EVs to transport under hypoxic conditions also gives them the opportunity to serve as a novel drug carrier in ischemic diseases^[57]^. GATA-4-containing EVs isolated from BMSCs induced the differentiation of BMSCs into cardiomyocyte-like cells, reduced hypoxia-induced apoptosis, and improved myocardial function. Concentroro-pretreated MSC-derived miR-1a-65p-containing EVs promote cardiac repair by targeting and reducing interleukin-1 receptor-associated kinase and inhibiting nuclear translocation of NF-κB p65, thereby protecting cells from hypoxic injury and providing superior therapeutic effects in anti-apoptosis and anti-inflammation^[58]^.

MSC-EVs also have a protective effect against myocardial injury caused by external stimuli. Human heart-resident mesenchymal progenitor cell EVs enriched with miR-146a-5p attenuate drug-induced oxidative stress in cardiomyocytes and their cardioprotective effects may be a novel approach for treating drug side effects^[59]^. Ischemia-pretreated MSCs target methyl CpG-binding protein 2 via miR-22-rich EVs to significantly reduce cardiac fibrosis, exert anti-apoptotic effects, and enhance stem cell protection for ischemic heart disease^[60]^. MSC-EVs also protect cardiomyocytes from adriamycin-induced cardiomyopathy by upregulating Survivin gene expression via the miR-199a-3p-Akt-specific protein 1/p53 signaling pathway^[61]^. MSC-EVs carrying cyclic RNATN4 interacted with miR-497-5p to upregulate MG53 expression in cardiomyocytes, significantly inhibited upregulated reactive oxygen species levels, reduced upregulated IL-1β, IL-6, and tumor necrosis factor-α levels, improved cell survival and inhibited apoptosis in cardiomyocytes, and attenuated sepsis-induced myocardial injury in rats with myocardial injury^[62]^.

In addition, Modified MSC-EVs may be especially effective in the treatment of heart disease. For instance, exposing hMSCs to titanium-surface-nanostructure pretreatment elevates EV secretion and facilitates the internalization of the hMSC-EVs by HUVECs. This promotes the migration and differentiation of the HUVECs, stimulating endothelial cells and cellular activity *in vitro* and possibly improving angiogenesis *in vivo*^[63]^. Similarly, EVs derived from MSCs doped with iron oxide nanoparticles induce a shift from an early inflammatory phase to a repair phase, reduce apoptosis and fibrosis, and enhance angiogenesis and the recovery of cardiac function^[64]^. hUC-MSC-EVs encapsulated in functional peptide hydrogels improve myocardial function by reducing inflammation, fibrosis and apoptosis and promoting angiogenesis^[65]^. Modified EVs provide a practical and effective method to perform myocardial regeneration.

### Kidney damage

In animal models, BMSC-EVs have been shown to promote recovery from kidney diseases such as chronic kidney disease and acute kidney injury (AKI)^[66,67]^. Thus, MSC-EVs may hold promise as therapeutic tools for kidney diseases. For instance, rats with unilateral ureteral obstruction represent a model of renal fibrosis in chronic kidney disease. Mechanical stress under unilateral ureteral obstruction induces nuclear expression of Yes-associated protein, which stimulates collagen deposition and interstitial fibrosis in the kidney. HUC-MSC-EVs ameliorate renal fibrosis by delivering casein kinase 1δ and E3 ubiquitin ligase β-transducin repeat-containing proteins to promote Yes-associated protein ubiquitination and degradation^[68]^. HUC-MSC-EVs also inhibit renal tubular epithelial cell apoptosis and reduce renal fibrosis by inhibiting the reactive oxygen species-mediated activation of the p38 mitogen-activated protein kinase/extracellular signal-regulated kinase 1/2 pathway^[69]^. miR-186-5p in MSC-EVs binds to the 3'-UTR of Smad5 and thus down-regulates Smad5 expression, attenuating renal injury/fibrosis in a unilateral ureteral obstruction model by inhibiting ECM protein aggregation and epithelial-mesenchymal transition^[70]^.

Adipose tissue-derived MSC-EVs have a positive effect on AKI^[71]^. MSC-EV yields were increased in three-dimensional culture compared to two-dimensional culture. The resulting MSC-EVs were also more efficiently absorbed by renal tubular epithelial cells, as evidenced by improved renal function, attenuated tubular pathological changes, reduced inflammatory factors, and suppressed T-cell and macrophage infiltration, significantly alleviating cisplatin-induced murine AKI^[72]^. Moreover, hUC-MSC-EVs reduce IL-1 receptor-associated kinase expression and inhibit NF-κB activity by upregulating miR-146b levels, thereby alleviating sepsis-associated AKI and improving survival in septic mice^[73]^.

### Osteoarthritis

In this section, we will discuss the regenerative repair capabilities of BMSC-EVs in orthopedic diseases. MSC-EVs improve the bone microenvironment and inhibit bone metastasis, representing a promising novel therapeutic for the prevention of bone and joint diseases and disease rehabilitation, with potential as a diagnostic and therapeutic tool for aging-related degenerative diseases^[74,75]^. MSC-derived EVs have successfully been used as cell-free therapies to guide cartilage differentiation in adipose-derived stem cells^[76]^. Transgenic MSCs overexpress bone morphogenetic protein 2, and their EVs can effectively function as growth factors to enhance specific bone regeneration *in vivo*^[77]^. In an ischemic limb mouse model, MSC-EVs enriched with VEGF protein enhance angiogenesis, perhaps by promoting high expression of VEGFR1 and VEGFR2 in endothelial cells. MSC-EVs activate receptors to affect angiogenesis and may play an important role in osteoarticular repair^[78]^. In addition, systemic administration of hucMSC-EVs highly enriched for the osteoprotegerin CLEC11A prevents bone loss and maintains bone strength in osteoporotic mice by enhancing bone formation, decreasing bone marrow fat accumulation, and reducing bone resorption^[79]^. MSC-EVs-CD73 may mediate cartilage repair and regeneration by activating adenosine through AKT and ERK signaling pathways, which in turn enhances cell proliferation, attenuates apoptosis, and modulates immune responses^[80]^. Moreover, MSC-EVs possessing the neutral endopeptidase CD10 (enkephalinase) exhibit immunomodulatory properties, effectively mitigating inflammatory activation of synoviocytes and cartilage degradation, thereby preserving cartilage homeostasis^[81]^. Therefore, the various bioactive factors carried by MSC-EV have potential in orthopedic diseases.

MSC-derived EVs cultured in a three-dimensional environment have the potential to induce improved repair. For instance, bone repair can be induced via lyophilization of optimized BMSC-EVs immobilized in a layered scaffold through the Bmpr2/Acvr2b competitive receptor-activated Smad pathway^[82]^. Combinations of human-induced pluripotent MSC-EVs and tricalcium phosphate scaffolds significantly altered the expression of gene networks involved in the PI3K/Akt signaling pathway and enhanced the proliferation, migration, and osteogenic differentiation of hBMSCs^[83]^.

In contrast, MSC-EVs carrying miRNA-128-3p from aged rats suppressed Smad5 and attenuated cellular osteogenic differentiation and fracture healing *in vivo*^[84]^. This suggests that MSC-EVs may also have an inhibitory role. Further research into these bioactive factors is needed.

## MSC-EVS AS CARRIERS FOR TARGETING TUMORS

### Colorectal cancer

Colorectal cancer (CRC), occurring in the colon and/or rectum, is a common cancer with a poor prognosis. There is growing evidence that EVs isolated from various MSC sources can have an inhibitory effect on CRC progression. For instance, the overexpression of miR-16-5p in BMSC-EVs inhibits CRC tumor cell progression through the downregulation of integrin α2^[85]^.

Intestinal inflammation leads to the abnormal secretion of growth and inflammatory cytokines that are closely associated with the onset and progression of CRC. EVs released from MSCs have anti-inflammatory effects and can repair tissue damage^[86]^. In a mouse model of dextran sodium sulfate-induced inflammatory bowel disease (IBD), hUC-MSC-EVs alleviated IBD by inhibiting neddylation through miR-326, suppressing the activation of the NF-κB signaling pathway, and reducing the production of inflammatory factors. The therapeutic effect of hUC-MSC-EVs with high miR-326 expression on IBD mice was significantly stronger than that of regular hUC-MSC-EVs^[87]^. In addition, hUC-MSC-EVs may function by regulating ubiquitin modification to restore the structural integrity of tissues in IBD mice^[88]^. Three-dimensional culture-generated MSC-EVs enriched with miR-1246 inhibited Nfat5 expression to mediate Th17 cell polarization, restored Th17/Treg homeostasis in inflamed periodontal tissue, and attenuated IBD^[89]^. Therefore, the therapeutic effect of MSCs on CRC may be attributed to EVs, which are rich in tumor-suppressor miRNAs. The use of MSC-EVs containing suppressor miRNAs to treat patients with CRC may become an effective approach in future clinical practice.

### Head and neck squamous cell carcinoma

Head and neck squamous cell carcinoma (HNSCC) is a general term for malignant tumors occurring in the squamous epithelial cells of the head and neck region. HNSCC is a common malignant tumor without clinically significant precancerous lesions; thus, most patients receive diagnoses at advanced stages^[90]^. Presently, HNSCC is managed through surgical procedures or radiation therapy, but the survival rates remain low, with high rates of cancer recurrence. Patients with recurrent and metastatic HNSCC face bleak prognoses, necessitating improved molecular diagnostics and effective treatments for better outcomes.

Dysregulated miRNAs are secreted within EVs and play a crucial role in the complex tumor microenvironment^[91]^. MSC-EVs recruited into the tumor microenvironment of oral squamous cell carcinoma significantly enhance HUVEC migration, invasion, and tube formation capacity by increasing matrix metalloproteinase 1 levels, thereby promoting tumor growth^[92]^. Thus, EVs provide a potential avenue for the treatment of HNSCC.

**Figure 3:**
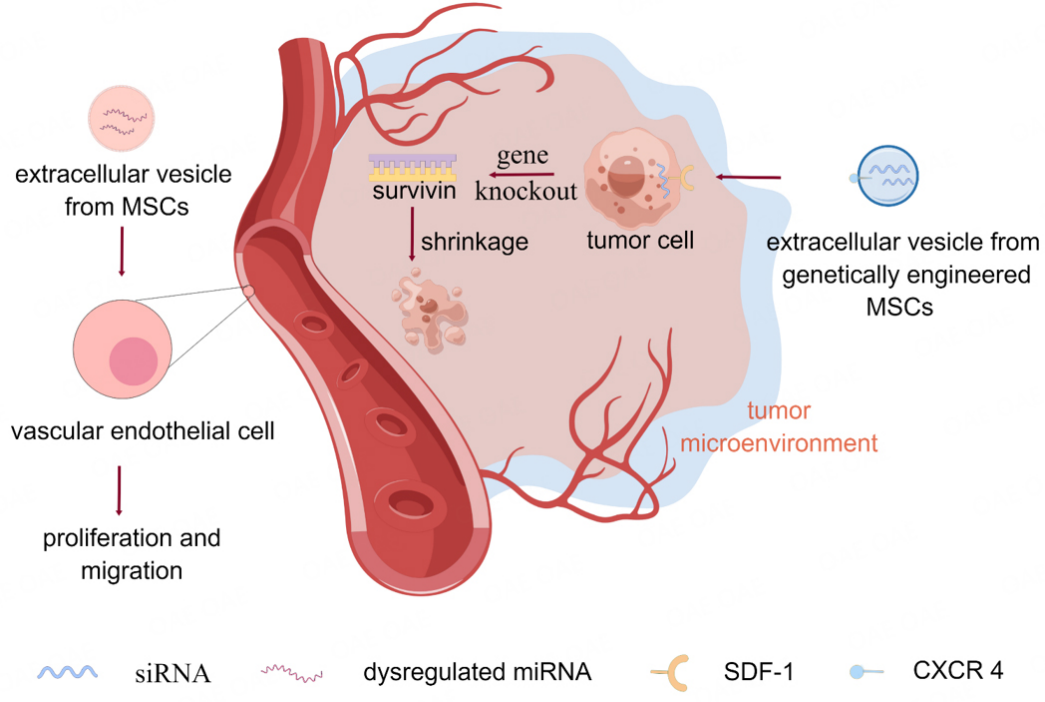
MSC-Exos play different roles in the tumor microenvironment by carrying different biological factors. MSC-Exos recruited in the OSCC tumor microenvironment promotes tumor growth by increasing MMP1 levels and promoting HUVEC migration, invasion, and tube formation capabilities. Genetically engineered MSC-Exos with high expression levels of CXC chemokine receptor type 4 (CXCR 4) specifically bind to stromal cell-derived factor 1 (SDF-1) on the tumor surface. The Exos efficiently accumulate at tumor sites and release their small interfering RNA (siRNA) into the tumor cells, knocking down the Survivin gene in tumor cells to inhibit tumor growth.

MSC-EVs can be employed as delivery vehicles to target tumors and surmount intra-tumor barriers. For example, MSC-EVs encapsulated with gold nanoparticles demonstrated the capability to penetrate and distribute throughout tumor tissues and cells in a model of head and neck cancer^[93]^. In HNSCC, miR-101-3p-enriched human BMSC-EVs inhibit the proliferation and tumor growth of oral cancer cells *in vitro* and *in vivo* by targeting the collagen type X alpha 1 chain gene^[94]^. Transgenic dental pulp MSCs can secrete EVs rich in therapeutic miRNAs, making the application of EV-based gene delivery vectors feasible^[95]^. Genetically engineered MSCs were employed to generate EVs with high expression levels of CXC chemokine receptor type 4 as a vector for targeted gene-drug delivery. CXC chemokine receptor 4 specifically binds stromal cell-derived factor 1 on the tumor surface. EVs carry small interfering ribonucleic acids (siRNA), which accumulate at the tumor site and enter the tumor cells to knock down the Survivin gene, thus inhibiting tumor growth *in vivo*^[96]^ [Figure 3]. This gene-drug delivery system has the potential for clinical application, providing a new approach to drug therapy for tumors.

## REGENERATIVE ROLE OF MSC-EVS IN NEUROLOGICAL DISEASES

The limited regenerative capacity of the nervous system constrains its ability to repair damage, necessitating the identification of novel treatments for neurological damage and the alleviation of neurodegenerative diseases. Stem cell EVs protect NSC-34 cells from oxidative damage and enhance cell viability, implying a potential role for stem cell EVs in motor neuron diseases^[97]^. MSC-EVs enhance rotational performance and attenuate neuropathology, encompassing Purkinje cell loss, cerebellar myelin loss, and neuroinflammation^[98]^. Thus, MSC-EVs offer promising applications for the treatment of neurological diseases.

This section discusses how MSC-EVs play a role in neurological injury. MSC-EVs promote normal myelin formation, increase mature oligodendrocyte and neuronal cell counts, and significantly enhance learning in preterm perinatal brain-injured animals^[99]^. MSCs mitigate ischemia-reperfusion injury in mouse brains by suppressing CDK6 expression through extracellular vesicle miR-26a-5p, inhibiting microglia apoptosis^[100]^. Cell-free preparations containing neuroprotective MSC-EVs can replace MSCs in the treatment of preterm infants with hypoxic-ischemic brain injury, thus avoiding the potential risks associated with the systemic administration of live cells^[101]^. Therefore, MSC-EVs may represent a novel strategy for the treatment of neurological sequelae following hypoxic-ischemic injury in preterm brains.

Modified EVs have demonstrated increased therapeutic effects to some extent. For example, gold nanoparticles (as markers of MSC-EVs) can selectively target pathologically relevant mouse model brain regions, where MSC-EVs are taken up by neuronal cells for therapeutic and targeted drug delivery^[102]^. Iron oxide nanoparticles can enhance angiogenesis and reduce inflammation and apoptosis in the damaged spinal cord, thereby improving spinal cord function during spinal cord injury treatment^[103]^. Additionally, exercise and BMSC-EVs exert synergistic effects on neuronal apoptosis and synaptic axonal remodeling in rats, inducing reductions in neuronal apoptosis, cerebral infarct volume, synapse formation, axonal regeneration, and significant recovery of neurological function^[104]^. Future studies aimed at maximizing the positive effects of MSC-EVs in the repair of nervous system pathologies are warranted.

### Alzheimer's disease

Alzheimer's disease (AD) is a neurodegenerative disorder characterized by a progressive decline in cognitive function. MSC-EVs contain various miRNAs that exert beneficial effects by mediating various factors/molecules and activating signaling cascades to regulate multiple genes, thus reducing neuropathological changes in neurological diseases^[105,106]^. MSC-EVs also carry neurotrophic factors and signaling modulators that may serve as potential therapeutic agents for neurodegenerative diseases^[107,108]^. The utilization of EVs represents a new direction for identifying novel therapeutic targets for AD.

Intranasally administered MSC-EVs improved AD-like phenotypes in a clinical mouse model^[109]^. Therefore, cell-free MSC-EV-based therapy stands as a promising therapeutic strategy for AD. BMSC-EVs reduced Aβ deposition and promoted cognitive recovery in AD mice by enhancing the expression of sphingosine kinase 1 and sphingosine 1-phosphate 1, thereby improving spatial learning and memory in mice^[110,111]^. Additionally, GDF-15-containing BMSC-EVs upregulated enkephalinase and insulin-degrading enzyme expression through the AKT/GSK-3β/β-catenin axis, attenuated Aβ42-induced apoptosis and inflammation in SH-SY5Y cells, and enhanced cell viability^[112]^.

Moreover, the presence of brain amyloid-β (Aβ) and specific tau proteome aggregates in the nervous system constitutes an important feature in patients with AD. Reducing Aβ accumulation may represent a potential treatment mechanism for AD^[113]^. EVs appear to have the ability to reduce Aβ accumulation and tau hyperphosphorylation, as well as transfer neuroprotective substances between neuronal cells^[114]^. For instance, UC-MSCs-EVs reduce neuronal apoptosis and promote neurological recovery in rats by inhibiting the activation of microglia and astrocytes^[115]^. EVs derived from human amniotic fluid MSCs attenuate microglia-induced inflammatory damage, thereby significantly reducing neurotoxicity and slowing AD progression^[116]^. Therefore, MSCs -EVs hold promise for the treatment of AD.

### Parkinson's disease

Parkinson's disease (PD) is a progressive degenerative neurological disorder characterized by the loss of melanin-containing neurons in the substantia nigra and other pigmented nuclei of the brainstem. MSCs can differentiate into dopaminergic neurons and produce neurotrophic substances, making MSCs and their EVs potential candidates for PD treatment^[117]^. hUC-MSC-EVs traverse the blood-brain barrier to reach the substantia nigra, alleviate apomorphine-induced asymmetric rotation, reduce nigrostriatal dopaminergic neuronal loss and apoptosis, upregulate dopamine levels in the striatum, stimulate SH-SY5Y cell proliferation, and inhibit apoptosis by inducing autophagy for repair in PD models^[118]^. Therapeutic peroxidase mRNA delivery from EVs designed with the EVtic device attenuates neurotoxicity and neuroinflammation in a PD model^[119]^. This suggests that MSC-EVs offer a novel approach for the treatment of PD.

## CONCLUSION

Despite the theoretical ability of MSCs to differentiate into multiple human cell types, in fact, most cell types are difficult to generate. Even though some differentiated cells are functionally comparable to normal human cells, uncertainty remains about their orientation. Additionally, MSCs are difficult to isolate and obtain, and their sources are constrained by ethical concerns. The direct injection of stem cells into hosts is associated with significant risks, including teratoma and tumor formation, massive graft cell death, vascular blockage, and immune responses. In contrast, EV treatment is promising and may be free from these risks. MSC-EV can not only achieve the therapeutic effects of MSCs but are also free from ethical issues and possess advantages such as weak immunogenicity and reduced risks of tumorigenic effects. While EVs have short half-lives and require repeated treatments, they also circumvent the risks of necrosis associated with graft death, which can lead to inflammation and vascular obstruction.

This report summarizes the utility of MSC-EVs. In brief, MSC-EVs have anti-inflammatory capabilities for treating rheumatoid arthritis, liver injury, COVID-19, and immune system disorders. They employ tissue-repairing capabilities for treating heart disease, kidney injury, and osteoarticular disorders. They serve as carriers to deliver tumor-suppressing factors to target tumor cells in colorectal and head and neck squamous cell carcinomas. Furthermore, they possess regenerative effects for the treatment of neurodegenerative disorders such as Alzheimer's disease and Parkinson's disease.

Moreover, the role of MSC-EV in many other intractable diseases is under constant experimentation. In immune dysregulation diseases, topical application of MSC-EV inhibits complement activation in the stratum corneum, thereby reducing neutrophil aggregation and its release of IL-17 and relieving psoriatic-like inflammation^[120]^. MSCs-EV-mediated miRNA-125b is used to attenuate desiccation syndrome in a rat model by targeting PRDM1 and inhibiting plasma cells, reducing inflammatory infiltration and restoring salivary secretion from salivary glands^[121]^. Additionally, MSC-EV promotes follicular development and improved ovarian activity in a rat model of premature ovarian failure^[122]^. The miR-369-3p carried by human amniotic fluid MSC-EV could inhibit the apoptosis of ovarian granulosa cells and exert therapeutic effects on premature ovarian failure^[123]^. MSC-EV-siRNA may alleviate the developmental progression of atherosclerosis and coronary heart disease by promoting cholesterol efflux and inhibiting intracellular lipid accumulation^[124]^. The stage of pancreatic cancer was significantly correlated with the low expression levels of miRNA-1231 in the peripheral blood extracellular vesicles. By injection of BM-MSC EV-miR-1231, tumor cell proliferation, migration, invasion, and adhesion were inhibited in mice. These results suggest that EV-miR-1231 could be a potential drug and diagnostic indicator for cancer therapy^[125]^. MSC-EV also has applications in other neurological disorders. MSC-EV improves a rat model of cavernous nerve injury by alleviating cavernous smooth muscle apoptosis^[126]^. In the experimental rat model, thermosensitive hydrogels containing adipose MSC-EVs were injected into the damaged tissues. The experimental results showed that EVs were retained and slowly released in the target tissue, effectively treating erectile disorders caused by cavernous nerve injury accompanied by radical pelvic surgery^[127]^. In addition, Intranasal administration of MSCs-EV holds promise for improving symptoms in autism-like mice. This finding provides new ideas and methods for the clinical treatment of such diseases^[128,129]^. In a mouse psoriasis model, topical application of MSC-EVs suppresses C5b9 complement complex through CD59 and reduces IL-17 secreted by neutrophils, thereby alleviating psoriatic skin inflammation^[130]^. In a sepsis mouse model, EVs produced by MSC by significant upregulation of IL-10 generation, inhibit the entry of cytokines into the systemic circulation and reduce the infiltration of neutrophils and monocytes, showing an anti-sepsis effect^[131]^. MSC-EVs contribute to regenerating muscle fibers to enhance myogenesis and hold promise for treating muscle diseases such as muscular dystrophy^[132]^. Intrathecal injection of MSC-EVs may alleviate suprapubic mechanical anomalous pain and urinary frequency in rats with interstitial cystitis by inhibiting the activation of NLRP3 inflammatory vesicles and TLR4/NF-κB signaling pathway, suppressing the activation of neuroglia and attenuating neuroinflammation in the dorsal horn of the spinal cord^[133]^. The potential application of MSC-EV in more intractable diseases is ongoing.

MSC-EV-based therapies hold promise, but several challenges remain^[134]^. First, the safety and efficacy of extracellular vesicles remain to be validated, and not all EVs exert positive effects. For example, bone regeneration was reduced in type 1 diabetes-derived extracellular vesicles compared to normal rat BMSC-EVs^[135]^. Therefore, selecting EVs that perform the correct function is a major challenge. Second, due to their relatively novel nature and limited technological advancements, EVs are difficult to extract. Extracted EVs often have low yields, function monofunctionally, possess short effective maintenance times, and are challenging to store and transport, limiting their clinical translation. Again, most of the current studies on EVs are in the preclinical animal testing stage, and there is still a considerable distance from the real clinical application, and the relevance of the relevant mechanisms remains to be verified. Moreover, while human embryonic MSC-derived conditioned media could rescue kidney function in rats with diagnosed chronic kidney disease, MSC-conditioned medium-derived EVs tested in the same experimental setting showed no protective effects on the kidney^[136]^. MSC-EV antigen CD73 did not exert extracellular 5'-nucleotidase activity in a mouse model of aGVHD and failed to ameliorate disease symptoms. EVs did not show the immunomodulatory potency of MSCs^[137]^. Consequently, whether the role of MSCs is realized through EVs remains somewhat controversial. In addition, it has been reported that EV-carried miRNAs have difficulty in fusing with cell membranes to deliver their cargoes, resulting in a failure to induce functional changes in target cells. This suggests that it is controversial whether EV-carried miRNAs act as effectors for intercellular communication^[138]^. Therefore, in clinical treatment, the advantages and disadvantages of EV therapy should be reasonably evaluated and integrated with the actual situation of individual patients.

With the continuous development of stem cell-related research and technologies, EVs could become a crucial therapeutic tool. However, preclinical experiments are not effective in determining clinical outcomes. In the face of many challenges, more research is urgently needed to promote the clinical application of EVs^[139]^. Large-scale mass production of EVs is a problem, which requires us to develop new techniques to optimize the culture conditions and increase the production volume. Secondly, it is worth studying how to separate the target EVs correctly. We need to explore a method that is both simple and efficient and maintains the integrity of EV biological efficacy. Targeted delivery of EVs to specific cells remains a problem. The discovery of specific receptors may contribute to improving EV targeting. The most important thing is to ensure the safety of EVs, which requires extensive clinical trials and long-term studies.

In addition, the biological characterization of the MSC-EV product during clinical translation must be reproducible and meet the requirements of the potency assay^[5]^. The results of existing studies show that the properties and potency of MSC-EV products depend on the MSC source and culture conditions: for example, 2 D and 3 D MSC culture types, *etc*. These different manufacturing parameters produce MSC-EVs with different therapeutic effects for different diseases. Therefore, it is critical to optimize the manufacturing process of human MSC-EV products with the desired properties to ensure quality control, reproducibility, and potency assays before clinical application for the expected disease targets. At the same time, measures should be taken to mitigate the impact of tissue source and donor on the reproducibility of biological activity and therapeutic efficacy of MSC-EV formulations. However, there is considerable uncertainty about this effect. Many clinical trials have shown that effective MSCs will produce therapeutically effective MSC-EVs. The composition of EVs released from transplanted MSCs *in vivo* may be different from that of EVs released from the same cells *in vitro*. Therefore, how to select therapeutically effective MSCs still needs further research. In addition, the very complex mechanism of action of MSC-EVs carrying different biological factors in the treatment of diseases is related to their spatiotemporal biodistribution, which requires the exploration of sensitive and specific markers to track their spatiotemporal biodistribution. In addition, the dose, route of administration, carrier molecule, and site of action of MSC-EVs are also related to the mechanism by which MSC-EVs work, and thus longer testing and technological advances are needed.

## DECLARATIONS

### Copyright

© The Author(s) 2024.
